# Attitudes toward inter-hospital electronic patient record exchange: discrepancies among physicians, medical record staff, and patients

**DOI:** 10.1186/s12913-015-0896-y

**Published:** 2015-07-12

**Authors:** Jong-Yi Wang, Hsiao-Yun Ho, Jen-De Chen, Sinkuo Chai, Chih-Jaan Tai, Yung-Fu Chen

**Affiliations:** Department of Health Services Administration, China Medical University, Taichung, Taiwan; Taichung Veterans General Hospital, Taichung, Taiwan; National Changhua University of Education, Changhua, Taiwan; Department of Otolaryngology, China Medical University Hospital, Taichung, Taiwan; Department of Healthcare Administration and Institute of Biomedical Engineering and Material Science, Central Taiwan University of Science and Technology, Taichung, Taiwan, No. 666, Buzih Road, Beitun District, Taichung, 40601 Taiwan

**Keywords:** Electronic patient record (EPR), System implementation, Information exchange, Privacy protection, User support

## Abstract

**Background:**

In this era of ubiquitous information, patient record exchange among hospitals still has technological and individual barriers including resistance to information sharing. Most research on user attitudes has been limited to one type of user or aspect. Because few analyses of attitudes toward electronic patient records (EPRs) have been conducted, understanding the attitudes among different users in multiple aspects is crucial to user acceptance. This proof-of-concept study investigated the attitudes of users toward the inter-hospital EPR exchange system implemented nationwide and focused on discrepant behavioral intentions among three user groups.

**Methods:**

The system was designed by combining a Health Level 7-based protocol, object-relational mapping, and other medical informatics techniques to ensure interoperability in realizing patient-centered practices. After implementation, three user-specific questionnaires for physicians, medical record staff, and patients were administered, with a 70 % response rate. The instrument showed favorable convergent construct validity and internal consistency reliability. Two dependent variables were applied: the attitudes toward privacy and support. Independent variables comprised personal characteristics, work characteristics, human aspects, and technology aspects. Major statistical methods included exploratory factor analysis and general linear model.

**Results:**

The results from 379 respondents indicated that the patients highly agreed with privacy protection by their consent and support for EPRs, whereas the physicians remained conservative toward both. Medical record staff was ranked in the middle among the three groups. The three user groups demonstrated discrepant intentions toward privacy protection and support. Experience of computer use, level of concerns, usefulness of functions, and specifically, reason to use electronic medical records and number of outpatient visits were significantly associated with the perceptions. Overall, four categories of independent variables were associated with the mean difference in the perceptions.

**Conclusions:**

Discrepant attitudes toward privacy and support among the three user groups are identified. Patients may require further education and communication regarding the system. Culturally fit e-Consent should be incorporated into the system to fully utilize the computing power of the Internet when also considering workload. The concern for misuse of EPRs might lead to low support among physicians. Highly readable EPR documents and managerial incentives for information exchange may improve system use.

## Background

Patient-centered health care cannot be accomplished without using electronic patient records (EPRs) that integrate fragmented patient records scattered over health care organizations (HCOs). EPRs enable health professionals to practice by exchanging clinical records from various sources that are specific to an individual patient. Exchanges among HCOs are expected to produce highly comprehensive patient-centered clinical information [1], leading to increased efficiency and quality of care. In addition, effective EPR exchanges enable the goal of continual care. Noteworthily, this study conformed to the terminology regarding the five stages of health care information systems (HIS) in the development of the electronic health record (EHR) proposed by the Medical Records Institute [2]. Electronic medical records (EMRs, third stage) are medical records completely in digital format from one single-provider source, whereas EPRs (fourth stage) further involve the electronic exchange of patient clinical information among HCOs.

In this era of ubiquitous information, however, technical, organizational, and individual barriers still preclude a uniform infrastructure for exchanging patient records over the Internet [3]. Prior research has proved that disseminating acquired knowledge in an organization is a major challenge because of employees’ resistance [4]. Exchanging patient records among different HCOs is a predictably considerable challenge. Eliminating the barriers to establish a collaborative platform of exchange requires considerable efforts including constructing the protocols and understanding the attitudes among different participants for designing supplementary measures that advance system use [5].

Investigating the attitudes of users in the process of EPR implementation is pivotal to user acceptance [6]. Studies have reported users’ attitudes toward EMRs from several perspectives. Physicians and nurses involved in developing EMR valued the functionality of the system but were concerned about time consumption and care quality [7]. In addition, experience in computer use contributed to the differences in the two concerns [1, 8, 9], and computer self-efficacy has been proven to be a strong predictor of the attitude toward information technology (IT) acceptance in hospitals [10]. Based on existing literature [11], the enforcement of security and privacy is a fundamental step in the development of EPRs because security and privacy are the major concerns of users. Concerns among users might still persist even though encryption and authorization have been adopted in EPRs. Physicians and nurses were concerned about the confidentiality of patient information and exhibited significant differences in their attitudes toward the impact on work [7]. However, another study determined that most physicians and patients believed that health information exchange would improve the quality of care [12]. Prior research has shown that 96 % of patients participating in medical record sharing were satisfied with their involvement in the treatment because of improved communication; however, suspicion regarding privacy concerns remained [13]. The aforementioned studies indicated that privacy, data security, health care quality, and work impact are the pivotal concerns of different types of user.

To promote the project of “Constructing the patient-centered environment of inter-organizational electronic patient record exchange,” the Department of Health (DOH) authorized the Taiwan Association for Medical Informatics (TAMI) to hold several public hearings. Excerpts of the hearings from the case hospitals in 2010 are presented as follows:

“Due to insufficient medical knowledge among patients, the content of medical records might cause misunderstanding and also possibly *inappropriate disclosure* of medical information.”

“If the content of electronic patient records in a USB flash drive *leaked out* intentionally or unintentionally, what will happen? It seems that *data security and privacy* are quite important.”

Security, privacy, and liability are clearly their primary concerns.

An evaluation of an HIS facilitates transforming the system into a highly effective advantage that supports health care delivery. Yusof, Papazafeiropoulou, Paul, and Stergioulas [14] categorized HIS evaluation research into three distinct domains: organizational, human, and technology. On the basis of this HIS evaluation framework, the current study assessed the following domains: the effect on patient care/work practice of the organizational domain, user-friendliness/attitude of the human domain [15, 16], and functionality/IT adaptation of the technology domain. Most research on user attitudes has been limited to one type of user or aspect. Seeing a dearth of analysis of various users of EPRs by using a comparative approach, this proof-of-concept study involved technologically implementing an inter-hospital EPR nationwide exchange system and managerially evaluating the users’ attitudes toward EPRs with a highlight of discrepant behavioral intentions among the three major user groups of the system, namely physicians, medical record staff (MRS), and patients. In addition, factors associated with the attitudes were investigated. In this study, the three user groups were hypothesized to differ in the level of EPR acceptance associated with diverse concerns. To our knowledge, research pertaining to the attitudes of MRS toward EPRs has yet to be conducted. This study was conducted in the context of implementing an EPR system.

## Methods

### Design of EPR

**Fig. 1:**
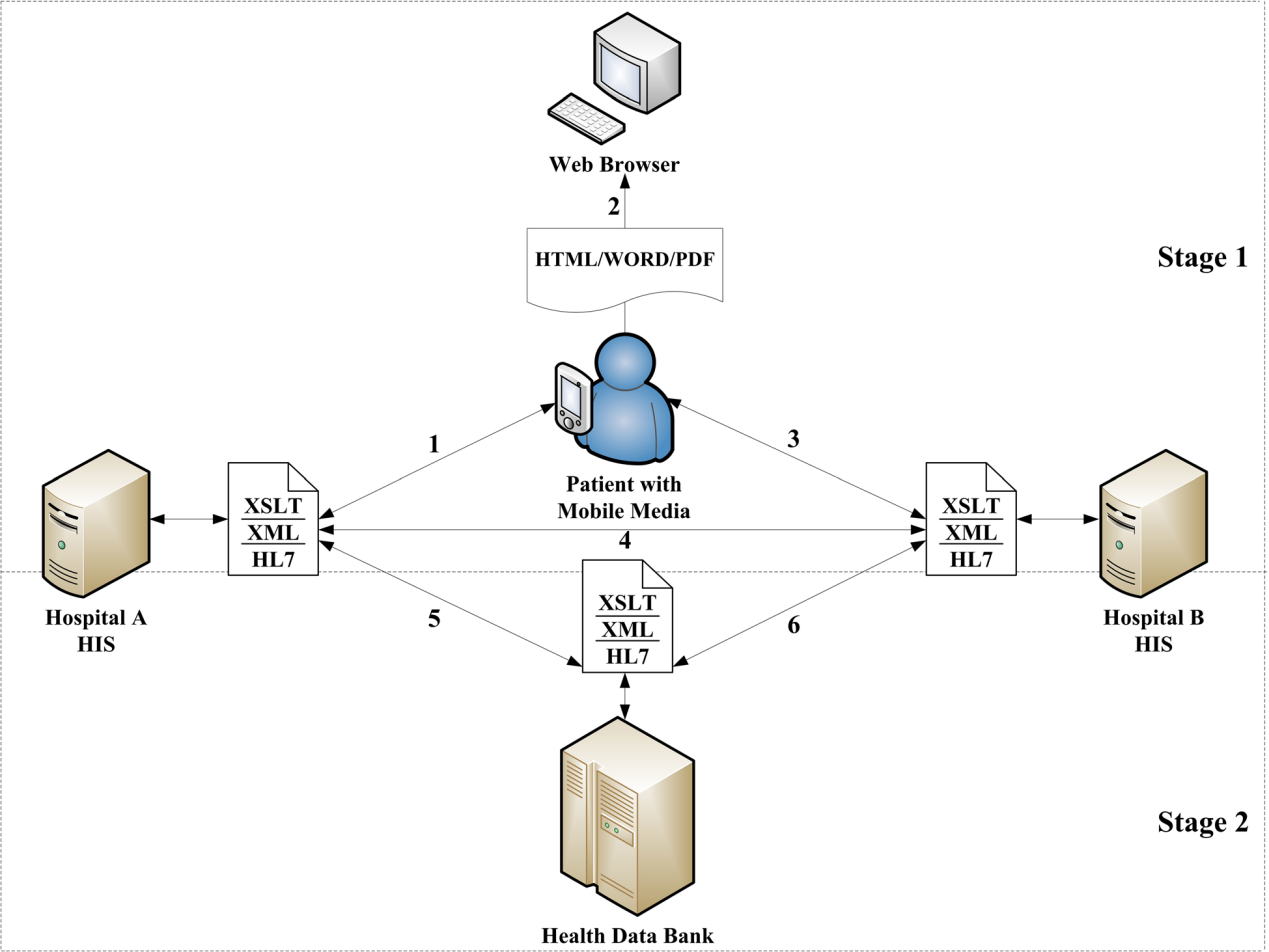
A concise conceptual infrastructure - data flow in object-relational mapping

The TAMI established the Taiwan Electronic Medical Record Template (TMT), a national standard that parallels Health Level 7 (HL7) Version 2.5 but was designed specifically to meet the requirements for exchanging clinical content encoded in Chinese. By conforming to the same TMT protocol, 11 hospitals that participated in this pilot plan could exchange clinical information with one another. A brief conceptual infrastructure conceived by the TAMI is illustrated in Fig. 1. This study contributes to EPR implementation by mapping data fields of patient records and verifying HL7. The EPR data flow is subsequently described to visualize the link between operational procedures and the aforementioned potentially relevant concerns of users.

The nationwide exchange plan consists of two stages. The first stage enables a patient, after submitting an application to Hospital A in the custody of patient records, to store and manage his or her medical records in a portable storage device (Path 1) and view the content by using a Web browser (Path 2). This stage enables a physician in Hospital B to add content into the same device (Path 3). Path 4 enables the two hospitals to share medical information. The second stage advances exchange by enabling medical information to be directly accessed by hospitals via the Health Data Bank, a dedicated server (Paths 5 and 6). Therefore, this stage might be named *Cloud Computing-based Healthcare Information System*, or simply *Health Cloud*, and applies the computing power of Internet technology.

In addition to using the TMT, the case hospital had to convert clinical content stored in a local EMR format into a format that was semantically interoperable among all platforms. Consequently, the patient-oriented user interface was incorporated with object-relational mapping (ORM) and Web-based medical informatics that acquired clinical data from various hospital information systems. ORM is a programming technique for converting data in incompatible formats, with metadata describing the mapping among objects in clinical databases [17]. This study adopted the technique of Extensible Markup Language (XML), which was embedded into the exported content of medical records, ensuring interoperability among patients or hospitals [18]. Through Extensible Stylesheet Language Transformations, a declarative XML-based language, a new document may be created using a processor in standard XML syntax or in another format such as PDF or HTML [19]. Users can read medical records by selecting different file formats, enabling optimal access on Web pages. In addition, instead of a generic electronic signature, encrypted digital signature and timestamp technologies, serving as a verification seal, are issued by the Healthcare Certification Authority whenever requested by the case hospital in generating an XML document. Encrypted XML documents with a timestamp enforce data security requirements legitimately.

Conceptually, the system was designed by the collaboration of the database, management, presentation, and acquisition layers and implemented to ensure interconnection, interoperability, transparency, and readability in a patient-centered, database-driven, technology-assisted, and management-oriented manner. EPRs support all clinical services in the ambulatory environments among the three user groups, namely outpatients, physicians, and MRS.

### Evaluation of the attitudes among users

#### Data source and measurement instrument

This study adopted a cross-sectional design and surveyed participants mainly at two metropolitan teaching hospitals located in central Taiwan, supplemented with other data sources. Because physicians, MRS, and patients have different viewpoints, three versions of a structured questionnaire were designed. Among the 25 items of the questionnaire, only three critical questions pertaining to privacy concerns and personal support for EPRs were common to all users. The ABC model proposes that an attitudinal assessment consists of affective, behavioral, and cognitive components [20, 21]. Therefore, the concerns of unfavorable effects, perceived usefulness of functions, perception of expected benefits, and behavioral intentions were incorporated into the instrument in this study. Three principal axis factor analyses with Varimax rotation were performed, and three components were extracted for each individual group, accounting for 52.32 %, 72.34 %, and 65.42 % of the variance of the user-specific questionnaires for physicians, MRS, and patients, respectively. All factor loadings of the items for every component in the three questionnaires were higher than 0.46, 0.63, and 0.48, which met the standards of convergent validity in construct validity [22, 23]. Extracted factors were named by examining the content meaning of every item in the same factor in each user-specific questionnaire. Despite different items in each user-specific questionnaire, identical components were found for all three questionnaires, namely Concern, Function, and (behavioral) Intention (to use EPR), as presented in Table 1. For the physician questionnaire, Items 1, 6, 7, 11, and 14 were extracted as the Concern component; Items 4 and 5 as the Function component; and Items 2, 3, 8, 9, 10, 12, 13, and 15 as the Intention component on the basis of the results of exploratory factor analysis (EFA). Regarding the questionnaire for the MRS, EFA revealed that Items 1, 6, and 7 were grouped as Concern; Items 4 and 5 were grouped as Function; and Items 2, 3, 16, 17, 18, and 19 were grouped as Intention. In the third factor analysis for the patient questionnaire, Items 1, 24, and 25 were extracted as the Concern component; Items 20 and 21 as the Function component; and Items 2, 3, 9, 10, 22, and 23 as the Intention component. Furthermore, the items identified as the same components by using EFA can be averaged or summated to indicate the components [23, 24].

**Table 1:** Attitudes among three types of user toward the implementation of inter-hospital EPR exchange, descriptive statistics, factor analyses, and Bivariate GLM test (25 comprehensive questionnaire items of the attitudes)

Items (in a scale of 1 ~ 5; 1 = disagree, 5 = agree)		Extracted components	Physician		Medical record staff		Patient	
			Mean	SE	Mean	SE	Mean	SE
	*Privacy of patient may be violated* ^a^	Concern ^g^	3.85	1.05	3.68	1.16	3.82	0.94
	*Should protect privacy through the conditional implementation by consent of patient* ^b ***^	Intention ^h^	3.91	1.03	4.11	0.86	4.49	0.76
	*Supporting the promotion of inter-hospital electronic patient records exchange* ^c **^	Intention	3.68	1.05	3.79	0.92	4.01	0.84
	User interface may affect the willingness to use the System ^d ***^	Function ^i^	4.17	0.95	3.25	1.21		
	Comprehensive functions will increase the willingness to use the System ^e^	Function	4.12	0.93	4.18	0.82		
	The exchange system may have the data security problem	Concern	3.94	1.05	4.04	1.04		
	Handling the exchange data increases work load	Concern	3.32	1.18	3.18	1.12		
	Would like to share my orders with physicians in other hospitals	Intention	3.64	0.97				
	Expect the System to provide more comprehensive patient records	Intention	3.79	0.95			3.93	0.82
	Can increase quality of health care ^f **^	Intention	3.63	0.99			3.89	0.78
	Can change habit of handwriting in medical records to avoid misunderstanding	Concern	3.19	1.11				
	Can reduce repeated lab tests	Intention	3.65	1.10				
	Can increase efficiency of diagnosis and treatment	Intention	3.41	1.14				
	Concern about patient’s potential misunderstanding for the content of medical records	Concern	3.86	1.03				
	Will benefit patient through continuing health care	Intention	3.79	0.91				
	Will bring convenience to medical records operation	Intention			3.82	0.77		
	Will save storage space for medical records	Intention			3.86	0.80		
	May save manpower	Intention			3.46	1.14		
	May increase efficiency of medical records management	Intention			3.61	0.88		
	Understand the function of electronic patient records exchange	Function					2.62	1.00
	May improve physician and patient relationship	Function					3.80	0.88
	Do not care about the leak of personal medical information	Intention					2.29	1.36
	Do not mind if physicians obtain my information through electronic exchange in case of a medical need	Intention					3.59	1.05
	Exchanging hospitals will confidentially protect patient information	Concern					3.69	1.13
	Electronic patient records provided by this system are reliable and safe	Concern					4.44	0.80

^a^ Total mean = 3.82, SD = 1.00

^b^ Total mean = 4.23, SD = 0.93. Types of user and gender showed significant between-group differences (*p* <0.001, *p* = 0.009, respectively): Patient > medical record staff and physician

^c^ Total mean = 3.85, SD = 0.95. Types of user showed significant between-group differences (*p* = 0.005): Patient > physician

^d^ Types of user, gender, and age showed significant between-group differences (*p* <0.001, *p* = 0.410, *p* <0.001, respectively): physician > medical record staff

^e^ Experience of computer use showed significant between-group differences (*p* = 0.021)

^f^ Types of user, gender, and age showed significant between-group differences in this result (*p* = 0.005, *p* = 0.030, *p* = 0.001, respectively): Patient > physician

^g^ Concerns for EPR

^h^ Behavioral intention to use EPR

^i^ Perceived usefulness of functions and features

^**^ *p* <0.01, ^***^ *p* <0.001 among type of user

An expert panel composed of two physicians, three senior system analysts, and one experienced supervisor in the medical records department evaluated the content validity of the three questionnaires. The research team revised the questionnaires according to their professional opinions before conducting the pilot survey. The reliability was obtained using Cronbach’s alpha for internal consistency [25]. After formal administration of the survey, answers to the questions showed adequate internal consistency (Cronbach’s alpha = 0.85, 0.80, and 0.72 for the physician, MRS, and patient versions, respectively) [26, 27]. The psychometric properties confirmed that the three questionnaires were effective instruments for assessing attitudes toward EPRs.

#### Definition of variables

The attitudes variables were scored on a 5-point Likert scale, in which 1 indicates *highly disagree* and 5 indicates *highly agree*. Only items with a significant mean difference among users served as primary dependent variables, which were Items 2 (privacy protection by patient consent) and 3 (supporting the promotion), for further multivariate analysis. Due to the fact that the survey was conducted in the introductory period of the EPR system, evaluation on actual organizational impacts may not be suitable. Instead, the user-perceived impacts were adopted.

The technology acceptance model (TAM) suggested that perceived usefulness of functions is a determinant of user acceptance [16]. Thus, this study applied the perceived usefulness of functions and features as an independent variable (Function, Table 1). As mentioned, the concerns for EPRs, including privacy violation, misunderstanding of the content, data security, and work load, were employed as an independent variable (Concern, Table 1) in predicting the two attitudes (Items 2 and 3). Both Concern and Function were coded dichotomously as low (< mean) and high (≧ mean) according to the requirement of a categorical independent variable by using general linear model (GLM), an effective method of estimating mean values in a multivariate model. Because the two dependent variables were identified as the Intention component (Table 1), the independent variables did not contain any other items in Intention to eliminate inevitable collinearity.

**Fig. 2:**
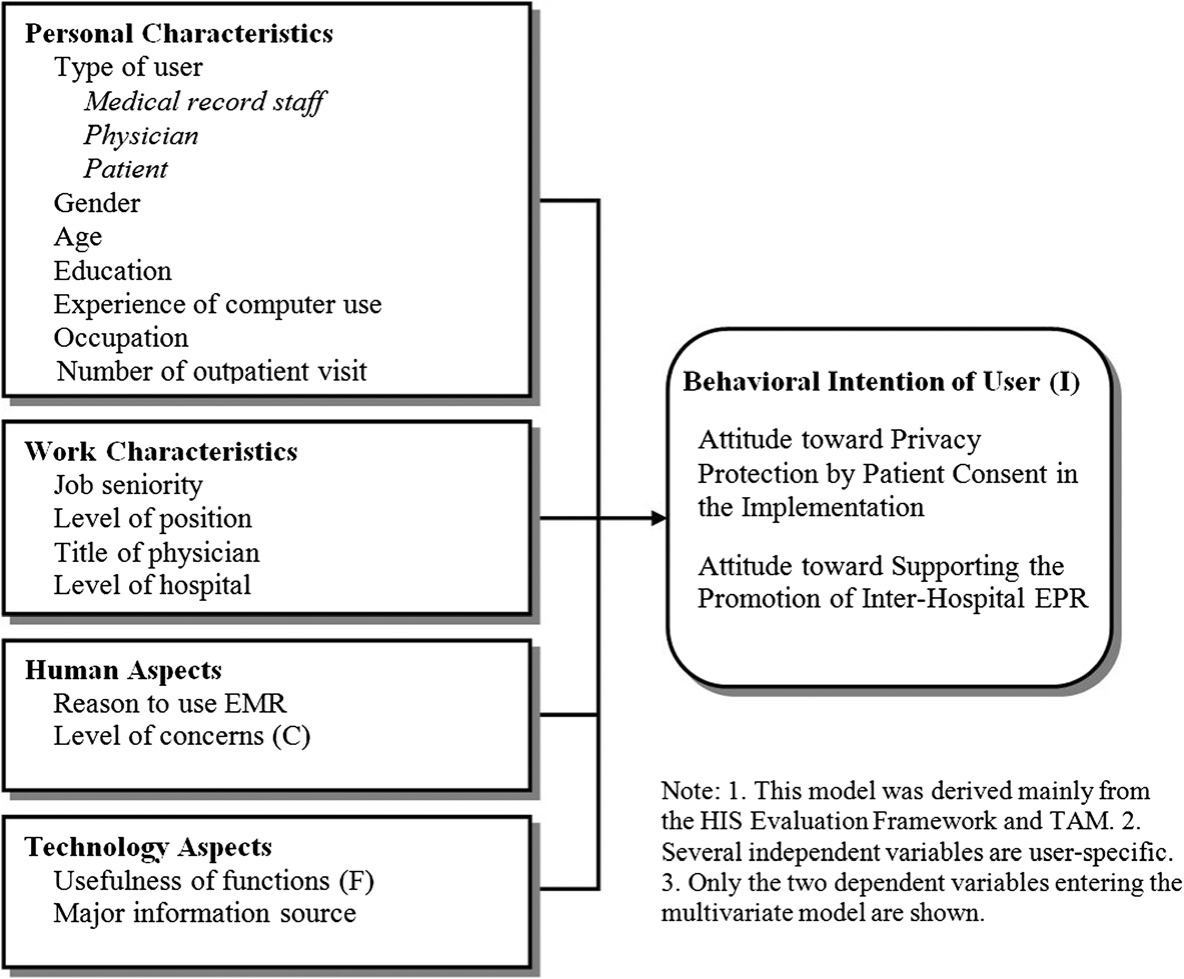
The UAMEPR

Computer experiences are closely related to HIS use, as mentioned previously. Thus, years of computer use was adopted as its measure in this study. Other common demographic variables, including type of user, gender, age, and education, served as associated factors for the attitudes toward HIS use, which have been suggested in previous studies [28, 29]. Because occupational and organizational characteristics might be associated with the attitudinal measures [7–9], positions, seniority, type of occupation, and hospital level were included in this study. Type of occupation was categorized on the basis of the Standard Industrial Classification System in Taiwan [30]. Categories exhibiting a low frequency were merged. Level of position was dichotomized as supervisor versus non-supervisor. Title of physician was classified as attending physician and resident. In addition, because EMRs are a predecessor to EPRs in the continuity of the staged HIS developments [2], the reason for using EMRs may be linked to the attitudes toward EPRs. Furthermore, the number of outpatient visits indicating health condition may be associated with the attitudes among patients [29] because frequent care users are more likely to demand the accumulation of their medical records among HCOs. The number of outpatient visits was categorized by quartile from the frequency distributions. Major information source was regarded as an associated factor because research has suggested that information source is an essential determinant of attitudes toward health policy [31, 32], and because fostering the EPR system is a progressive health policy of the Taiwan government. Previous studies have revealed that hospital level may be related to the attitude of hospital personnel toward information sharing and system adoption [33, 34]. To compare the scores of the attitudes among the three major user groups, the variable “type of user” was created to enable between-group comparisons. In addition to type of user, four user-common independent variables, including gender, age, education, and experience of computer use, were entered in the adjusted between-group analyses for Items 2 and 3. All the variables were assessed in a self-reported manner, except for hospital level, which was directly adopted from the official classification of the DOH [35]. The independent variables in the full model were classified into personal characteristics, work characteristics, human aspects, and technology aspects. These factors, which are known to influence the outcome variables, were held constant in multivariate analysis. The conceptual framework, namely the user acceptance model of EPR (UAMEPR), is shown in Fig. 2. This model was derived from the HIS evaluation framework and the TAM and incorporates the characteristics of the different types of user (physician, MRS, and patient) with the distinct aspects (organization, human, and technology) involving various extracted components of attitude (Concern, Function, and Intention).

#### Participants

From March 2010 to May 2010, nearly 3 months after the first stage of EPR was launched in the case hospital (a tertiary medical center of all specialties consisting of more than 2000 beds), respondents were selected using a mixed sampling method in this study. Physicians were selected from a table of random numbers by using a simple random sampling method. All MRS at the medical center were asked to complete the survey. Patients were selected according to voluntary participation through a convenience sampling method because the sampling frame of existing and potential patients was not obtainable. The scenarios from which the three types of respondent were sampled are detailed as follows:

1. Physicians participating in the explanation meetings for EPRs or their regular meetings at two teaching hospitals (one medical center and one regional hospital);
2. MRS at their working site at one medical center;
3. Patients who joined the promotional meetings for EPRs at one medical center (92), supplemented with persons in two government organizations (33), two private companies (40), and one university (31). Various sources for the patients sample might advance its representativeness for the population. The patients recruited from all sources did not play an active role in the system development, and thus, regarding them as patients is appropriate.

Finally, the survey obtained 379 valid samples: 155 physicians, 28 MRS, and 196 patients. Most of the participants can be regarded as potential users [36]. The sample size was statistically determined according to the equations of the attitudinal scale on the basis of previous research [6, 7, 37]. The proposed research was approved by the institutional review board of China Medical University, Tawian, under No. DMR97-IRB-016. All of the questionnaires administered requested the voluntary participants to sign informed consent forms. Participation in this survey was completely anonymous.

#### Statistical analysis

Factor analysis was performed to identify the structural components underlying the 25 attitudinal items. After the components of attitudes toward EPRs were examined using EFA, the subsequent statistical approach focused on differentiating the behavioral intentions among the three groups of users by using GLM. GLM was used in estimating the least squares means for each level of the independent variables and testing mean differences in the scores of the nine common response questions (Items 1–7, 9–10, Table 1). GLM is suitable for an unbalanced design [38, 39] and can substantially show predicted means while adjusting for all other variables [40]. This study performed multivariate GLM with post hoc test to determine user differences in attitudes (Items 2 and 3). The attitudes were compared among and within all user groups. In addition, collinearity diagnostics was conducted using indices including tolerance, variance inflation (VIF), condition index (CI), and eigenvalue (λ). Data were analyzed using SAS Version 9.2.

## Results

The overall response rate for the questionnaires was 70 % (58 %, 70 %, and 82 % for physicians, MRS, and patients, respectively). The demographic characteristics are presented in Table 2. Other characteristics of the physicians not shown in the table include non-supervisors (83.87 %) and residents (54.84 %). More than half of the physicians (58.71 %) reported convenience in accessing medical information as the reason to use EMRs. Approximately two of five patients (42.35 %) reported two to five outpatient visits within the past 3 months. Nearly half of the patients (46.94 %) reported that they had never heard of EPRs. Among the four common independent variables, all three user groups similarly reported 10 or more years of experience in computer use as a majority within each group. However, gender, age, and education showed significant differences among the physician, MRS, and patient groups (all *p* <0.001).

**Table 2:** Characteristics of physicians, MRS, and patients

Variables	Total		Physician		Medical record staff		Patient	
	(*n* = 379)		(*n* = 155)		(*n* = 28)		(*n* = 196)	
	*n*	%	*N*	%	*n*	%	*n*	%
Personal Characteristics								
*Gender* ^a^								
Male	214	56.46	116	74.84	4	14.29	94	47.96
Female	165	43.54	39	25.16	24	85.71	102	52.04
*Age* ^a^								
≦25	55	14.51	--	--	7	25.00	48	24.49
26-35	184	48.55	93	60.00	13	46.43	77	38.29
36-45	82	21.64	40	25.81	5	17.86	38	19.39
46-55	38	10.03	18	11.61	2	7.14	18	9.18
56-64	14	3.69	4 ^c^	2.58	1	3.57	9	4.59
≧65	6	1.58	--	--	--	--	6	3.06
*Education* ^a^								
Junior high school or lower	9	2.37	--	--	--	--	9	4.59
Senior high school	25	6.60	--	--	9	32.14	16	8.16
College	282	74.41	125	80.65	19	67.86	138	70.41
Master degree	53	13.98	20	12.90	--	--	33	16.84
Doctoral degree	10	2.64	10	6.45	--	--	--	--
*Experience of computer use* ^b^								
Never or <1 year	13	3.43	3	1.94	1	3.57	9	4.59
1-3 years	18	4.75	7	4.52	1	3.57	10	5.10
4-6 years	56	14.78	16	10.32	7	25.00	33	16.84
7-10 years	90	23.75	33	21.29	8	28.57	49	25.00
≧10 years	202	53.30	96	61.94	11	39.29	95	48.47
*Occupation*								
Industry or commerce							26	13.27
Government or education							30	15.31
Healthcare							36	18.37
Service							38	19.39
Student							32	16.33
Other							34	17.35
*Number of outpatient visit in the past three months*								
0							48	24.49
1							53	27.04
2-5							83	42.35
≧6							12	6.12
Work Characteristics								
*Job seniority*								
<1 year			19	12.26	5	17.86		
1-5 years			61	39.35	7	25.00		
6-10 years			23	14.84	6	21.43		
11-15 years			24	15.48	6	21.43		
≧16 years			28	18.06	4 ^c^	14.29		
*Level of hospital*								
Medical center			105	67.74				
Regional hospital			50	32.26				
Human Aspects								
*Reason to use EMR*								
Convenient to access medical information			91	58.71				
Useful for work			11	7.10				
Not prefer handwriting			13	8.39				
EMR is mandatory			30	19.35				
No use and no benefit			5	3.23				
Other			5	3.23				
Technology Aspects								
*Major information source of this EPR*								
Friend or colleague							36	18.37
Internet							12	6.12
Government institution							8	4.08
Relative							5	2.55
Hospital							29	14.80
Other							14	7.14
Never heard of							92	46.94

^a^ Significant difference among the three types of user (*p* <0.001, Chi-square test)

^b^ No significant difference among the three types of user (*p* = 0.207, Chi-square test)

^c^ Categories with few sample numbers (<5) will be merged into other categories in the next levels of analysis to avoid unreliable estimations and to obtain comparability across type of user

Attitudinal scores for the 25 items are presented in Table 1. The mean of 4.23 in Item 2 indicated that, overall, users agreed that personal privacy should be protected by means of patient consent. Notably, patients reported insufficient knowledge of EPR exchange (Item 20, mean = 2.62), strongly disagreed with not caring about medical information leaking (Item 22, mean = 2.29), but regarded EPRs provided by the system as reliable and safe (Item 25, mean = 4.44). In unadjusted analyses, type of user was associated with significant mean differences in the four items (Items 2, 3, 4, and 10; bold). Of the three common items shared among all users, Items 2 and 3 showed significant mean differences (*p* <0.001 and *p* = 0.005, respectively). Physicians showed higher mean scores compared with MRS in the item of user interface affecting the willingness to use (*p* <0.001). Years of computer use was a significant factor for Item 5 (*p* <0.05). No signs of collinearity were observed.

Regarding the mean scores of the two constructs identified using factor analysis and used in UAMEPR, the physicians perceived usefulness of functions (F) higher (mean = 4.15) than the MRS and patients did (mean = 3.72 and 3.21, respectively). The physicians and MRS exhibited the same level of concern (C) for the system (both 3.63), whereas the patients showed the highest level of concern (3.76) among the three groups. The items for the constructs included user-specific and user-common questions, as shown in Table 1.

The factors associated with mean differences in the attitude toward privacy protection by patient consent in the implementation of EPRs are shown in Table 3. When all four user-common independent variables were held equal, type of user was significantly associated with the attitude toward this privacy concern (*p* <0.001, *R*^2^ = 0.1257). The patients reported higher scores (4.490) compared with both the MRS (4.058) and physicians (3.925), with a marked difference of 0.565 adjusted score between the patients and physicians (data not shown). Among the physicians, job seniority, reason to use EMR, level of concerns, and usefulness of functions were associated with significant mean differences. Among the MRS, gender, age, experience of computer use, job seniority, and level of concerns were associated with significant mean differences. Among the patients, age, level of concerns, and major information source of this EPR were significantly associated with attitude.

**Table 3:** Means of the attitude toward privacy protection by patient consent in the implementation of inter-hospital EPR exchange among users; multivariate GLM test (controlling for all other variables, total *n* = 379)

Variables ^d^	Physician ^a^		Medical record staff ^b^		Patient ^c^	
	(*n* = 155)		(*n* = 28)		(*n* = 196)	
	Mean	*P*	Mean	*P*	Mean	*P*
Personal Characteristics						
*Gender*		0.113		0.027^*^		0.232
Male	3.834		4.401		4.430	
Female	4.133		3.686		4.555	
*Age*		0.837		0.002^**^		0.041^*^
≦25			3.322		4.565	
26-35	3.807		4.603		4.516	
36-45 (≧36 for medical record staff)	4.141		4.205		4.512	
46-55	4.031				4.382	
56-64	3.514				4.309	
≧65					4.173	
*Experience of computer use*		0.252		0.011^*^		0.186
Never or <1 year					4.209	
1-3 years (≦3 years for physician)	3.724				4.442	
4-6 years (≦6 years for medical record staff)	3.815		3.740		4.459	
7-9 years	3.734		4.609		4.395	
≧10 years	4.005		3.782		4.592	
Work Characteristics						
*Job seniority*		0.017^*^		0.021^*^		
<1 year	3.414		4.401			
1-5 years	3.581		4.613			
6-10 years	4.085		3.424			
11-15 years (≧11 years for medical record staff)	4.490		3.736			
≧16 years	4.321					
*Human Aspects*						
*Reason to use EMR*		<0.001^***^				
Convenient to access medical information	4.089					
Useful for work	4.053					
Not prefer handwriting	3.943					
EMR is mandatory	3.539					
No use and no benefit	2.742					
Other	3.393					
*Level of concerns*		<0.001^***^		0.002^**^		<.001^***^
Low	3.644		3.522		4.177	
High	4.172		4.565		4.696	
*Technology Aspects*						
*Usefulness of functions*		0.012^*^		0.547		0.270
Low	3.725		3.956		4.410	
High	4.128		4.131		4.541	
*Major information source of this EPR*						0.017^*^
Friend or colleague					4.485	
Internet					4.701	
Government institution					3.801	
Relative					4.977	
Hospital					4.188	
Other					4.627	
Never heard of					4.582	

^a^ R^2^ = 0.2516

^b^ R^2^ = 0.8329

^c^ R^2^ = 0.1946

^d^ Only variables statistically significant are shown

^*^ *p* <0.05, ^**^ *p* <0.01, ^***^ *p* <0.001

The factors associated with supporting the promotion of inter-hospital EPR exchange are shown in Table 4. After other characteristics were adjusted, type of user was strongly linked to attitudinal support (*p* = 0.009, *R*^2^ = 0.1167). The patients reported the highest scores (3.928), followed by the physicians (3.587). However, mean differences between the patients and MRS as well as those between the physicians and MRS were both non-significant (data not shown). Among the physicians, age, hospital level, reason to use EMR, level of concerns, and usefulness of functions were associated with significant mean differences. Among the MRS, gender and age were associated with significant mean differences. Among the patients, experience of computer use, occupation, number of outpatient visits in the past 3 months, level of concerns, and major information sources of this EPR were significant predictors of the scores. Notably, people working in the health sector reported lower scores compared with those in the service industry.

**Table 4:** Means of the attitude toward supporting the promotion of inter-hospital EPR exchange among users; multivariate GLM test (controlling for all other variables, total *n* = 379)

Variables ^d^	Physician ^a^		Medical record staff ^b^		Patient ^c^	
	(*n* = 155)		(*n* = 28)		(*n* = 196)	
	Mean	*P*	Mean	*P*	Mean	*P*
Personal Characteristics						
*Gender*		0.171		0.021^*^		0.237
Male	3.618		4.822		4.426	
Female	3.904		3.692		4.559	
*Age*		0.034^*^		0.018^*^		0.109
≦25			3.533		3.951	
26-35	3.437		4.887		3.973	
36-45 (≧36 for medical record staff)	3.296		4.350		4.027	
46-55	4.542				4.097	
56-64	3.844				4.049	
≧65					4.372	
*Experience of computer use*		0.521		0.498		0.029^*^
Never or <1 year			4.093		3.367	
1-3 years (≦3 years for physician)	3.451		4.158		3.777	
4-6 years (≦6 years for medical record staff)	3.779		4.520		4.139	
7-9 years	3.857				4.097	
≧10 years	3.956				3.987	
*Occupation*						0.015^*^
Industry or commerce					4.129	
Government or education					4.279	
Healthcare					3.866	
Service					4.186	
Student					3.990	
Other					3.627	
*Number of outpatient visit in the past three months*						0.046^*^
0					3.832	
1					3.986	
2-5					4.067	
≧6					4.350	
Work Characteristics						
*Level of hospital*		0.006^**^				
Medical center	4.022					
Regional hospital	3.500					
*Human Aspects*						
*Reason to use EMR*		0.024^*^				
Convenient to access medical information	4.161					
Useful for work	4.122					
Not prefer handwriting	3.980					
EMR is mandatory	3.550					
No use and no benefit	2.870					
Other	3.995					
*Level of concerns*		0.010^*^		0.574		<0.001^***^
Low	3.889		4.042		3.655	
High	3.468		4.298		4.227	
*Technology Aspects*						
*Usefulness of functions*		0.004^**^		0.589		0.407
Low	3.458		4.048		4.077	
High	3.937		4.292		3.966	
*Major information source of this EPR*						0.049^*^
Friend or colleague					4.255	
Internet					3.964	
Government institution					3.757	
Relative					4.426	
Hospital					3.920	
Other					4.192	
Never heard of					3.910	

^a^ R^2^ = 0.2486

^b^ R^2^ = 0.6309

^c^ R^2^ = 0.1212

^d^ Only variables statistically significant are shown

^*^ *p* <0.05, ^**^ *p* <0.01, ^***^ *p* <0.001

## Discussion

### Discrepancies among the three user groups

The three user groups exhibited marked attitudinal differences in several survey items (Tables 1, 3, and 4). The patients tended to highly agree with privacy protection, system promotion, and quality increment through exchange, but reported insufficient knowledge of the exchange system. Contradictions justify the necessity of explaining the functionalities and advantages of the system. The physicians and MRS, however, reported a low consent to the corresponding items. Notably, the physicians expressed relatively conservative concerns about nearly all of the items except for user interface. The discrepant behavioral intentions identified at all levels of analysis require further attention. Furthermore, additional efforts are required to communicate the importance of EPRs to people working in the health industry.

Security and privacy concerns have been evaluated in previous research [41]. Considering the viewpoints of users is crucial for system success [8]. This study identified the characteristics of users whose intention of privacy protection by patient consent was low (Table 3). Among the user groups, age, job seniority, and level of concerns presented repeated differences in the intention of patient consent.

The patients exhibited higher attitudinal support compared with the physicians and MRS (Tables 3 and 4). The theory of reasoned action (TRA) and the theory of planned behavior (TPB) may assert that this attitude is a pivotal factor of the subsequent behavioral support and, accordingly, assist in tackling the low support problem with pragmatic solutions [42, 43]. In addition, social cognitive theory (SCT) [44, 45] and expectancy theory [46, 47] should be considered for designing effective reinforcements to encourage usage. This study speculates that, for example, further emphasis on capitation reimbursement and pay for performance (P4P) schemes may, in a financial incentive, motivate health care providers to collect additional comprehensive patient records for improved health outcomes and quality of care [48], if perceived usefulness of functions is high (TAM). Hence, targeting the characteristics of users who obtained low attitudinal scores is meaningfully a prelude to the subsequent strategic plan for motivating the use.

### Human aspects and technology aspects explaining the differences

Level of concerns proved to be a robust determinant of the discrepant intentions (Tables 3 and 4). All of the users who exhibited a low level of concerns expressed low levels of intention toward privacy protection by patient consent. Nevertheless, the result of this factor in predicting EPR support appeared to be inconsistent between the physicians and patients. The physicians exhibiting low concerns reported high attitudinal support. By contrast, the patients who reported high support were those who expressed high concerns. The high average score of 3.86 for Item 14, “Concerned about patient’s potential misunderstanding of the content of medical records” (Table 1) delineates the physicians’ reservation, which may have hindered their support for EPRs. Moreover, this result reflected the liability concern of clinical practitioners, as mentioned, which differs from that of patients. Furthermore, evidence suggested that the negative emotional reactions from the patients seemed to be minimal because EPR enhances communication and provides patients with a comprehensive understanding of their health condition [49] if EPR use is under the conditions of physicians-in-charge and not-for-profit [36]. In addition, an exported highly readable EPR document with standardized formats highlighting the most pertinent information should improve the perceived quality of documents [50], thus reducing this concern. The final point depends on the efforts of HIT.

The finding that perceived usefulness of functions is positively associated with agreeing to both privacy protection and the support of the exchange system among the physicians (Tables 3 and 4) is consistent with that of previous research [51]. User interface and comprehensiveness of function that both match the needs of users lead to increased acceptance of the system [52, 53]. The link between the reason to use EMR and perceptions of EPR indicated that the user attitude toward EMR might be a prerequisite in implementing the EPR system (Tables 3 and 4). Forcing the promotion of EPR on the physicians whose attitudes toward EMR is “no use and no benefit” is not feasible (Table 3). Thus, a stepwise promotion is recommended.

The discrepant intention of privacy protection by patient consent exclusively characterized differences in the standpoint between the physicians and patients (Table 3). The results indicated that the vulnerable state of patients predisposed them to seek privacy and confidentiality protection by informed consent [54] and suggested that additional efforts should be directed toward patient education, ensuring their right to informed consent. Conversely, the physicians might tend to empower themselves with EPRs without the absolute consent of patients when considering urgent clinical needs and quality of care (Items 10 and 15; Table 1). This phenomenon may relate to work control or the autonomy of physicians [51, 55].

Regarding the patient consent concern, which is crucial to successful EPR implementation, e-Consent might be a solution [56]. The patients tended to agree with sharing their medical information with other hospitals in case of a medical need (Item 23, 3.59; Table 1), but highly expressed privacy to be protected by their consent (Table 3). Correspondingly, Coiera and Clarke [56] proposed a variety of e-Consents including general consent, general consent with specific denial, general denial with specific consent, and general denial. Several points must be addressed in this study. First, to solve the reservation concerns, e-Consent needs to be incorporated into the system *before* the full implementation. Second, the four aforementioned prototypes might not fit all nations because research has suggested attitudinal differences between the general public in the United States and Japan in their electronic medical records handling [36]. Failure to meet the privacy requirements of patients may lead to undesirable consequences [54]. Hence, the discrepant perceptions of privacy merit further ethical research on need assessment of electronic informed consent in a culturally appropriate manner. Finally, Safran and Goldberg [3] suggested that “sometimes high tech can also produce high touch,” and a balance [56] between a culturally fit e-Consent and work load accounts for successful implementation.

The finding that the patients with a high use of care express high support may be explained by the frequent physician-seeking behaviors, particularly among patients with high levels of illness seeking further advice [57]. Logically, healthy individuals might not necessarily perceive a need for sharing medical information with other physicians in a different location.

### Identifying the differences among users on the road to health cloud

This study contributes to the existing literature in several substantive aspects. First, the concept of patient-centered practices was actualized using ORM and the computing power of the Internet. Second, this study addressed the discrepant behavioral intentions toward EPRs among the users, which was named *The Phenomenon of Difference in Indifference*. Third, the characteristics associated with differences in the intentions were identified. Notably, reason to use EMR and number of outpatient visits were two distinctive factors. Fourth, physicians may be more concerned with patient misunderstanding and usefulness of function out of their liability for care. The patients perceived the system more positively but lacked sufficient knowledge of the exact EPR functions. The MRS scored in the middle of the three groups in attitudes and tended to be more concerned with work-related concerns. By combining the third and fourth points, *The Phenomenon of Difference in Difference* may be the terms. Finally, an EPR-specific model of evaluation, UAMEPR, was proposed in this study and can be used in future research.

The status quo of the first stage EPR implementation in the case hospital is worth noting. Within the first year of the pilot test, more than 1300 patients experienced applying their EPRs in a dedicated counter for which MRS are responsible. The exchange functionalities were enabled among the 11 participating HCOs in the pilot plan. However, the first stage of EPR is currently pending in Taiwan until the second stage, Health Cloud, is established. Again, the general public conveyed their concerns regarding privacy leaking immediately after the authorities announced the incoming plan of the second stage at which only a paper-based consent will be adopted [58]. Paper-based consent may lead to an additional work burden because of frequent human contact [3]. Future research is necessary to examine paper-based consent, e-consent, and e-consent with customized options.

Certain limitations might have potentially biased the findings. First, some questionnaire items are specific to a single type of user. Even though these items were identified using EFA and categorized in the same component names (e.g., Concern), a comparison between types of user for this component may bias the inferential statistics in an unknown direction. However, multivariate analyses for all user groups and for each user group may revamp this reservation. Second, because of the small sample size of the MRS, the statistical testing power may have been attenuated. However, the sample size still accounts for over 70 % of the staff members in the medical record department of the case hospital. Third, some samples were selected from a single site and may thus not fully represent its population. Finally, all survey scores were based on self-report, thus biasing the findings conservatively.

## Conclusion

Implementing an inter-organization EPR exchange system requires the radical incorporation of individual, organizational, legal, and ethic endeavors. This study revealed the discrepant behavioral intentions among three user groups, with the lowest support from the physicians and the highest support from the patients. Policy makers should target the associated characteristics to design culturally appropriate interventions incorporating the implementation of the *Cloud Computing-based Healthcare Information System.* Several suggested related theories and managerial incentives may be applied for advancing the intention to use.

Information overload depicts our era. However, information sharing and acquisition is a transaction cost [59, 60] in which some health care providers may not decide to invest. As a fundamental peculiarity in health care [61], asymmetric information remains in this Internet age and continues to affect the effectiveness and efficiency of care [62]. To deliver improved care and to enhance patient benefits, systematic research that examines the effective schemes of promoting medical information sharing and exchange involving IT, behavioral and social sciences, and other disciplines should be prioritized.
